# Research of postpartum endometritis in Japanese Black cattle with cystic ovarian disease by vaginal mucus test and endometrial cytology

**DOI:** 10.5194/aab-63-1-2020

**Published:** 2020-01-07

**Authors:** Naoki Yamamoto, Ryo Nishimura, Yosuke Gunji, Mitsugu Hishinuma

**Affiliations:** 1 NOSAI Shimane, 105 Tonomachi, Matsue, Shimane 690-0887, Japan; 2 United Graduate School of Veterinary Science, Yamaguchi University, 1677-1 Yoshida, Yamaguchi 753-8515, Japan; 3 Laboratory of Theriogenology, Joint Department of Veterinary Medicine, Faculty of Agriculture, Tottori University, 4-101 Koyama-Minami, Tottori 680-8553, Japan; 4 NOSAI Tottori, 271 Higachisono, Hokuei town, Tohaku district, Tottori 689-2202, Japan; a current address: Meat Inspection Center, Shimane Prefectural Government, 1677-2 Senyama, Asayama town, Ooda, Shimane 699-2212, Japan

## Abstract

The relationship between endometritis and cystic ovarian disease (COD) is still unclear in Japanese Black cattle. Endometritis is classified into clinical endometritis (CE) and subclinical endometritis (SE). The objective of this study was to clarify the interaction between postpartum endometritis (CE and SE) and COD in Japanese Black cattle. Twenty-six suckled cows with COD (COD group) and 16 suckled cows with cyclical ovarian activity (CA group) were submitted for the experiment. Uterine conditions of cows were classified into three groups (normal, CE, and SE) with vaginal mucus test and endometrial cytology. The combined data of CE and SE were represented as data for total endometritis (EMT total). The prevalence of EMT total in the COD group (42.3 %, 11/26) was significantly higher than that of the CA group (12.5 %, 2/16). The mean percentage of polymorphonuclear neutrophils (PMN %) in the COD group was significantly higher than that of the CA group at 40–60 DPP (days postpartum). Compared to 61–295 DPP, the mean PMN % at 40–60 DPP was significantly higher in the COD group. The diameters of uterine horn and cervix did not differ among normal uterine condition, CE and SE in the COD group, and they did not differ between normal uterine condition and SE in the CA group. However, endometrial thickness during both 40–60 and 61–295 DPP were greater in the COD group than in the CA group. In conclusion, Japanese Black cattle with COD have a potential implication on endometritis at 40–60 DPP compared to the normal ovarian cycle. As a specific symptom was not observed by transrectal ultrasonography, endometrial cytology is effective for diagnosis of SE in Japanese Black cattle.

## 1 Introduction

Cystic ovarian disease (COD) and endometritis are major reproductive problems in both dairy and beef cattle during the postpartum period, resulting in a prolonged open period (Garverick, 1997; Gobikrushanth et al., 2016). According to statistical information from the Ministry of Agriculture, Forestry and Fisheries of Japan in 2018 (http://www.maff.go.jp/j/chikusan/kikaku/lin/l_hosin/, last access: 4 January 2020), the mean number of breeding beef cows per farm in Japan is around 10, indicating that the impact of reproductive problems of individual cow is crucial for the financial status of the farm. An ovarian cyst is characterized by one or more follicular structures larger than 25 mm in diameter in the absence of a corpus luteum (CL) (Todoroki et al., 2001). Lack of a luteinizing hormone (LH) surge (Hamilton et al., 1995) and LH receptor development in follicles (Kawate, 2004) are major factors in the occurrence of COD in dairy cattle. However, the pathogenesis of COD is still unclear due to the complexity of the disorder and the heterogeneity of the clinical signs (Vanholder et al., 2006). Endometritis induces ovarian disorder, resulting in subfertility in dairy cattle (Herath et al., 2006, 2007; Williams et al., 2007). A relationship between endometritis and COD has been reported in dairy cattle (Kesler and Garverick, 1982; Bosu and Peter, 1987; Tsousis et al., 2009). The relationships among uterine infection, endotoxin production, and resumption of postpartum ovarian activity have been established (Mateus et al., 2003). Intrauterine infections are considered to increase the risk of cyst formation (Bosu and Peter, 1987; Kim et al., 2005). Endometrial cytology by the cytobrush method revealed that the percentage of polymorphonuclear neutrophils (PMN %) was high in dairy cattle with COD at 6 weeks postpartum (Senosy et al., 2011). However, these previous studies in dairy cattle cannot exclude the effects by milk production that cause puerperal problems (Opsomer and Kruif, 2009) and ovarian dysfunction (Opsomer et al., 2006) in high-yielding dairy cattle. It was assumed that Japanese Black cattle were less susceptible to effects by milk production on postpartum endometritis compared to dairy cattle because Japanese Black cattle had one-tenth of the total milk yield of dairy cattle during lactation (Shingu et al., 2002). Since postpartum endometritis is also common in Japan (Suzuki, 2012), similar mechanisms relating to endometritis are assumed to provoke COD in Japanese Black cattle, and we will evaluate the relationship between endometritis and COD without the effect of milk production. However, the relationship between endometritis and COD is still unclear in Japanese Black cattle. Therefore, the study of these two diseases in the postpartum period is necessary to enhance the reproductive performance of Japanese Black cattle.

In dairy cattle, endometritis can be classified into clinical endometritis (CE) and subclinical endometritis (SE) by vaginal mucus test and endometrial cytology (Sheldon et al., 2006; Pleticha et al., 2009; Senosy et al., 2009; Barański et al., 2013). Especially endometrial cytology is important for the diagnosis of SE (Polat et al., 2015; Pothmann et al., 2015). Endometrial cytology performed with the cytobrush (Kasimanickam et al., 2005; Barlund et al., 2008; Senosy et al., 2011) and uterine lavage techniques (Kasimanickam et al., 2005; Santos et al., 2009; Galvão et al., 2011; Cocchia et al., 2012) has been used to determine the PMN %. There is no study that evaluated the relationship between endometritis and COD with the vaginal mucus test by Metricheck and cytobrush method in Japanese Black cattle. Transrectal ultrasonography (US) has been widely accepted for reproductive examination. Although the diagnosis of SE by endometrial cytology and US has been reported in dairy cattle (Kasimanickam et al., 2004), the relationship between these methods has not been studied in Japanese Black cattle. Therefore, the incidence rate of endometritis with COD and efficiency of US for endometritis diagnosis should be examined in Japanese Black cattle.

In beef cattle, uterine involution is completed at 37.7–56.0 DPP (days postpartum) (Noakes et al., 2009). Uterine inflammation reaches a peak at 1–3 DPP and the prevalence of uterine disease decreased gradually with the extension of postpartum period (Sheldon et al., 2009). In Japanese Black cattle, correlation between uterine condition and DPP in Japanese Black cattle with COD has not been studied yet.

The objective of this study is to reveal the interaction between postpartum endometritis (CE and SE) and COD in Japanese Black cattle with vaginal mucus test and endometrial cytology. Diagnostic accuracy of US for endometritis was also examined.

## 2 Materials and methods

### 2.1 Ethics statement

Animal handling and experimental procedures were carried out by following the Guidelines for Proper Conduct of Animal Experiments by the Science Council of Japan (http://www.scj.go.jp/ja/info/kohyo/pdf/kohyo-20-k16-2e.pdf, last access: 4 January 2020).

### 2.2 Animals

A field trial was conducted during April 2014 to August 2016 on the 15 commercial farms in Okuizumo town, Shimane Prefecture, Japan. Herds of 1 to 100 cows were selected for the study. The cows with COD (COD group) were selected from cows that were requested for reproductive therapy because of anestrous after 40 DPP having a cystic structure larger than 25 mm in diameter at one side ovary or both ovaries. The cows with cyclical ovarian activity (CA group) were picked up randomly from cows with CL (diameter: 14–24 mm) or follicle (diameter: 10–25 mm) after 40 DPP without any reproductive therapy nor artificial insemination. Twenty-six suckled Japanese Black cows (age: 8.7±3.5 years old) between 41 and 286 DPP (mean±SD: 101.2±55.4 d) for COD group and 16 suckled Japanese Black cows (age: 5.1±2.9 years old) between 40 and 80 DPP (mean±SD: 50.9±14.0 d) for CA group were submitted for the experiment. Parities of suckled cows in COD and CA group was 6.7±2.9 and 3.5±2.4, respectively. The cows were subjected to standard management practices in each region, provided with fresh water, and fed with hay and concentrate. The cows were clinically normal, and their body condition scores (1 to 5 scales with 0.25 increment points, BCS) were between 2.5 and 3.5.

### 2.3 Experimental design

All cows received rectal palpation and transrectal US using a real-time B-mode scanner with a 7.5 MHz linear array transducer (Tringa linear, Esaote (Pie Medical), the Netherlands). The cross-sectional frozen images of the cervix, uterine horn at the base of the left and right uterine horn, and the diameter of the largest follicle of one side was captured. The diameters of cervix and both uterine horns and the endometrial thickness were measured on a personal computer (screen ruler professional, Melanto Ltd, Bulgaria) (Sugiura et al., 2018). The presence of uterine fluid was also examined by US as previously reported (Kasimanickam et al., 2004). Subsequently, vaginal mucus assessment and endometrial cytology were performed by Metricheck (Simcro Tech Ltd, Hamilton, New Zealand) (Pleticha et al., 2009; Senosy et al., 2009) and the cytobrush method (Kasimanickam et al., 2005; Sheldon et al., 2006; Barlund et al., 2008; Senosy et al., 2011), respectively.

### 2.4 Evaluation of vaginal mucus and endometrial cytology

The vaginal mucus was collected by Metricheck in a similar manner as in previous studies (Pleticha et al., 2009; Senosy et al., 2009). After the cytobrush assessment, the Metricheck device was inserted into the vagina. The cup of the device was inserted to external uterine orifice and subsequently the opposite side of the device was elevated slightly to fill the cup with vaginal mucus. Then the device was withdrawn gently from the vagina. Vaginal mucus was scored according to previous studies (Williams et al., 2005; Sheldon et al., 2006; Senosy et al., 2009) on a 0 to 3 scale (score 0 represents clear or translucent mucus; score 1 represents mucus containing flecks of white or off-white pus; score 2 represents discharge containing <50 % white or off-white mucopurulent material; and score 3 represents discharge containing ≥50 % purulent material, usually white or yellow, but occasionally sanguineous).

Endometrial cytology by the cytobrush method was performed according to previous studies (Kasimanickam et al., 2005; Sheldon et al., 2006; Senosy et al., 2011). Briefly, the rod of the cytobrush, made from stainless steel (51 cm long and 2 mm in diameter) was threaded on the top of the rod with nylon (25 mm long and 4 mm in diameter). The rod and brush parts were placed in a stainless-steel tube (50 cm long and 4 mm in diameter). The cytobrush was inserted into a disposable plastic sheath and covered with a sanitary plastic sleeve (IMV Technologies, L'Aigle, France). Before use, the cytobrush was sterilized with formaldehyde gas. The external parts of the organs of reproduction and their margins were cleaned with benzalkonium and alcohol before the cytobrush was inserted into the vagina. When the cytobrush was passed through the cervix and inserted into the base of the uterine body under rectal operation, the brush was exposed on the dorsal membrane of the uterine wall. Then, the endometrial samples were collected by rotating 360${}^{\circ }$ twice while in contact with the dorsal wall of the uterine wall. Cytology slides were prepared by rolling the cytobrush on three clean glass slides and immediately fixed with Cytokeep II (Nippon Shoji, Osaka, Japan). The slides were stained with Giemsa stain within 2 h after the preparation. To assess endometrial inflammation by PMN % in an objective way, cytological assessment was determined by counting a minimum of 200 cells at 400× magnification (Kasimanickam et al., 2004; Ahmadi et al., 2007; Senosy et al., 2009).

Pathological conditions of the uterus were allocated into three groups by scoring vaginal mucus (Sheldon et al., 2006) and PMN % (Barlund et al., 2008; Gobikrushanth et al., 2016). In this study, the cutoff value of PMN % was set at 8 % in accordance with previous reports which recommended 8 PMN % as a good diagnostic cutoff value to discriminate between normal and endometritis in dairy cattle (Barlund et al., 2008) and beef cattle (Salah and Yimer, 2017). A cow with a mucosal score less than 2 and PMN % less than 8 % was considered normal. A cow with a mucosal score above 2 was considered CE, whereas a cow with a mucosal score less than 2 and PMN % above 8 % was considered SE.

### 2.5 Statistical analyses

Morbidity with endometritis was analyzed by a one-side test with Fisher's exact test. PMN % and diameters of largest follicle, cervix, and uterus at the base of the horn among groups were analyzed with analysis of variance and Tukey's test. Differences with P<0.05 were considered significant. Analysis of 2×3 Fisher's exact test was performed with R software (The R Foundation for Statistical Computing, Vienna, Austria). The other data were analyzed using Ekuseru-Toukei 2008 software for Windows (SSRI, Tokyo, Japan).

**Table 1:** Prevalence of clinical endometritis (CE) and subclinical endometritis (SE) in Japanese Black cattle diagnosed with vaginal mucus test and endometrial cytology. In parentheses is the number of cows.

Days postpartum	Ovarian	Uterine condition		
(DPP)	condition	Normal${}^{a}$	CE	SE
40–60	COD${}^{b}$ (6)	33.3 (2)	16.7 (1)	50.0 (3)
	CA${}^{c}$ (12)	83.3 (10)	0.0 (0)	16.7 (2)
61–295	COD${}^{b}$ (20)	65.0 (13)	30.0 (6)	5.0 (1)
	CA${}^{c}$ (4)	100.0 (4)	0.0 (0)	0.0 (0)

${}^{a}$ Cows without clinical nor subclinical endometritis.${}^{b}$ Cows with cystic ovarian disease.${}^{c}$ Cows with cyclical ovarian activity.

**Table 2:** Diameter of uterine horn and cervix measured with transrectal US and PMN % assessed with endometrial cytology in Japanese Black cattle.

Days postpartum (DPP)	Parameters	Ovarian condition	
		COD	CA
40–60	Number of cows	6	12
	Diameter of uterine horn (mm)	23.5±6.9	22.0±2.7
	Diameter of cervix (mm)	34.0±6.3	30.9±3.8
	Endometrial thickness (mm)	$15.1\pm 0.9^{a}$	$10.9\pm 1.3^{b}$
	PMN %	$17.4\pm 14.9^{a,A}$	$4.8\pm 6.9^{b}$
61–295	Number of cows	20	4
	Diameter of uterine horn (mm)	20.0±3.7	21.3±1.5
	Diameter of cervix (mm)	31.3±6.2	31.4±4.7
	Endometrial thickness (mm)	$14.6\pm 2.3^{a}$	$10.1\pm 0.9^{b}$
	PMN %	$2.9\pm 3.0^{B}$	1.9±1.4

Mean±SD.${}^{a,b}$ Significantly different between ovarian conditions (P<0.01).${}^{A,B}$ Significantly different between DPP (P<0.01).

## 3 Results

The cows were allocated into 40–60 and 61–295 DPP groups, and the table shows the proportion of cows with normal uterus, CE and SE in COD group, and CA group in Table 1. The proportions of normal uterus, CE, and SE did not differ statistically between CA and COD groups at both 40–60 and 61–295 DPP. The combined data of CE and SE were represented as data for total endometritis (EMT total) in this study. The prevalence of EMT total of the COD group (42.3 %, 11/26) was significantly higher than that of the CA group (12.5 %, 2/16) (P<0.05). In the COD group, 66.7 % (4/6) at 40–60 DPP and 35.0 % (7/20) at 61–295 DPP of the cows were detected as EMT total. In the CA group, 16.7 % (2/12) at 40–60 DPP and 0.0 % (0/4) at 61–295 DPP of the cows were detected as EMT total. In the COD group, the prevalence of uterine fluid was as follows: normal uterus, 13.3 % (2/15); CE, 0.0 % (0/4); and SE, 0.0 % (0/7). In the CA group, uterine fluid was not observed in any uterine conditions of normal and SE. The diameter of uterine horn and cervix in COD group and CA group at 40–60 and 61–295 DPP are presented in Table 2. At both 40–60 and 61–295 DPP, there were no significant differences in diameters of uterus and cervix between COD and CA groups. The diameter of uterine horn and cervix did not differ among uterine conditions of normal, CE, and SE in the COD group, and they also did not differ between uterine conditions of normal and SE in the CA group. In both 40–60 and 61–295 DPP, the thicknesses of endometrium were significantly greater in the COD group than in the CA group (P<0.01) (Table 2). Mean PMN % of the COD group was significantly higher than that of the CA group at 40–60 DPP (P<0.01). Compared to 61–295 DPP, the mean PMN % of 40–60 DPP was significantly higher in the COD group (P<0.01) (Table 2).

## 4 Discussion

In dairy cattle, some studies reported the relationship between endometritis and COD in dairy cattle (Kesler and Garverick, 1982; Bosu and Peter, 1987; Kim et al., 2005); however, research into postpartum dairy cattle cannot exclude the effects of milk production. High milk production and the related negative energy balance increase the risks of endometritis (Opsomer and Kruif, 2009; Cheong et al., 2011) and ovarian dysfunction (Opsomer et al., 2006). In Japanese Black cattle, the prevalence of EMT total in the COD group was significantly higher than that of the CA group in this study. The mean PMN % in the COD group was also higher than that in the CA group at 40–60 DPP. This is the first report that reveals that Japanese Black cattle with COD carry a high risk for endometritis (CE and SE) at 40–60 DPP. The finding in Japanese Black cattle in the present study also suggests that not high milk production but physiological postpartum endometritis can contribute to the development of COD in the early postpartum period in cows because the effects by lactation in the postpartum period are smaller in Japanese Black cattle than in dairy cattle (Shingu et al., 2002). These results are similar to previous studies which reported the relationship between endometritis and COD in dairy cattle (Kesler and Garverick, 1982; Bosu and Peter, 1987; Kim et al., 2005). Several reasons for the relationship between COD and endometritis have been described in dairy cattle. Ovulation is an important factor for the incidence of COD, and endometritis is associated with failure of ovulation (Opsomer et al., 2000; Sheldon et al., 2002). *Escherichia coli* is one of the major pathogens of endometritis in dairy cattle (Sheldon et al., 2002; Williams et al., 2005; Westermann et al., 2010; Sun et al., 2011). Uterine infection by *E. coli* increased lipopolysaccharides (LPS) in ovarian follicles (Herath et al., 2007), which causes dysfunction of the ovulation mechanism and responsiveness for steroid hormone (Polat et al., 2015). LPS also suppresses the secretion of GnRH and LH, resulting in inhibition of ovulation in dairy cattle (Peter et al., 1989; Karsch et al., 2002; Magata et al., 2014). As *E. coli* is one of the major endometrial pathogens in beef cattle (Salah and Yimer, 2017) and dual-purpose cattle (Ricci et al., 2015), *E. coli* seems to cause endometritis in Japanese Black cattle. Postpartum uterine involution is completed around 40 DPP in Japanese Black cattle (Izaike et al., 1989). In other beef cattle, the prevalence rate of SE decreases after 50 DPP (Santos et al., 2009). Changes in PGF${}_{2\alpha }$ metabolite (PGFM) after calving reflect uterine involution in beef cattle (Vecchio et al., 1992). Though plasma concentration of PGFM increased to eliminate bacterial pathogens such as *Escherichia coli* and *Trueperella pyogenes* from uterine epithelium (Vecchio et al., 1994), a long-term effect of PGF${}_{2\alpha }$-induced COD in dairy cattle (Kaneko and Takagi, 2014; Bosu and Peter, 1987). Therefore, an increase in LPS and continuous PGF${}_{2\alpha }$ secretion by a uterus infected with pathogens such as *E. coli* and *T. pyogenes* in beef cattle (Salah and Yimer, 2017) can lead to COD in Japanese Black cattle.

Our results differ with a previous report that the prevalence of endometritis at 25 DPP had no effect on the existence of follicular cysts at 35 and 65 DPP in dairy cattle (Gobikrushanth et al., 2016). This might be due to the difference in research period of endometritis. Though Gobikrushanth et al. (2016) mentioned that the prevalence of endometritis was 71.4 % at 25 DPP, postpartum uterine recovery was not completed at 25 DPP in dairy cattle (Noakes et al., 2009) in which the uterus had endometritis physiologically (Sheldon et al., 2009).

One possible reason for the incidence of endometritis in cow with COD is dysfunction of the estrus cycle. In normal healthy cows, the uterus should be sterile by 6–8 weeks postpartum (Sheldon and Dobson, 2004). The occurrence of the estrus cycle after calving seems to be important for uterine involution and clearance (Noakes et al., 2009). However, in this study the prevalence of EMT total and mean PMN % in Japanese Black cattle with COD at 40–60 DPP was higher than that at 61–295 DPP. And there was no significant difference between COD and CA groups in PMN % at 61–295 DPP. If COD gives rise to endometritis, this uneven distribution of endometritis on DPP should not be observed. These results indicate that COD at 61–295 DPP has no effect on the occurrence of endometritis. Rather, the presence of follicular cysts may enhance uterine clearance by producing estradiol (Yoshioka et al., 1996), acting as a stimulator of the hormonal immune system (Grossman, 1985). Thus, it is conceivable that COD was not a cause of endometritis. As numerous factors are known to induce COD (Vanholder et al., 2006), COD at 61–295 DPP may be induced by the other reasons rather than endometritis.

In this study, however, the prevalence of EMT total did not differ between the COD group and CA group at neither 40–60 nor 61–295 DPP. The limitations of the diagnostic method for endometritis might have influenced the results. It has been reported that the distribution of PMN throughout the endometrium is different for each pathogen (Bonnett et al., 1991). And the results of PMN % by the cytobrush method increased with the presence of *T. pyogenes* but not with other bacterial pathogens, such as *E. coli* (Westermann et al., 2010). Thus, even though the cytobrush method had a high specificity to diagnose SE, its sensitivity is low or moderate (Pascottini et al., 2016). When bacteria were analyzed independently, the growth densities of *T. pyogenes*, *Proteus* spp., and *Fusobacterium necrophorum* were associated with the presence of mucopurulent or purulent vaginal mucus but not with other bacteria such as *E. coli* (Williams et al., 2005). Consequently, these reports raised the possibility that some of the cows diagnosed as endometritis negative were false negatives in this study. A possible reason for this is the situation that almost all cows received artificial insemination or reproductive treatment at 40–60 DPP in the experimental region, and the number of cows which received no treatment at 61–295 DPP in the CA group was small in this study.

Endometrial thicknesses at both 40–60 and 61–295 DPP were greater in the COD group in this study, and these results could be explained by the fact that estrogen induces edema in the endometrium and increases endometrial thickness (Sugiura et al., 2018). However, in the present study, we did not evaluate steroid hormones. In a further study, we should examine the levels of steroid hormones and the relationship between endometritis, COD, and steroid hormones. Neither CE nor SE had an effect on the diameter of uterus or cervix in this study. Moreover, the prevalence of uterine fluid by transrectal US disagreed with the result of vaginal mucus test by Metricheck and endometrial cytology by cytobrush. Other specific findings were not observed by transrectal US. Though it was reported that existence of uterine fluid by US was suitable for diagnosis of SE as endometrial cytology in dairy cattle (Kasimanickam et al., 2004), this study revealed that diagnosis only by US was not able to detect CE nor SE in Japanese Black cattle. Especially in a cow with COD, it is difficult to diagnose whether inflammation or estrogen is the cause for uterine fluid. As shown in several previous reports (Sheldon et al., 2006; Pleticha et al., 2009), vaginal mucus test and endometrial cytology are essential for the diagnosis of endometritis in cow with COD. However, vaginal mucus test is not a common diagnosis method for veterinarians in Japan. The cytobrush method in cows has been established during the last decade (Kasimanickam et al., 2004, 2005; Barlund et al., 2008), and few veterinarians use the cytobrush method to detect endometritis. Considering the risk for endometritis in cows with COD, vaginal mucus test and endometrial cytology should be carried out as a general diagnosis method at 40–60 DPP in Japanese Black cattle.

In conclusion, Japanese Black cattle with COD have a potential implication for endometritis at 40–60 DPP compared to the cows with a normal ovarian cycle. Thus, we recommend vaginal mucus test and the cytobrush method for examining endometritis when COD is diagnosed. Especially endometrial cytology is essential to diagnose SE in Japanese Black cattle.

## Data Availability

The original data are available upon request to the corresponding author.
